# A Joint Regional Analysis of Resistance Combinations in *Escherichia coli* in Humans and Different Food-Producing Animal Populations in Germany Between 2014 and 2017

**DOI:** 10.3389/fpubh.2022.823613

**Published:** 2022-06-09

**Authors:** Beneditta Suwono, Tim Eckmanns, Heike Kaspar, Bernd-Alois Tenhagen

**Affiliations:** ^1^Department Biological Safety, Unit Epidemiology, Zoonoses and Antimicrobial Resistance, German Federal Institute for Risk Assessment, Berlin, Germany; ^2^Department Infectious Disease Epidemiology, Unit Healthcare-Associated Infections, Surveillance for Antibiotic Resistance and Consumption, Robert Koch Institute, Berlin, Germany; ^3^Unit Antibiotic Resistance Monitoring, German Federal Office of Consumer Protection and Food Safety, Berlin, Germany

**Keywords:** antimicrobial resistance, *Escherichia coli*, regional analyses, surveillance and monitoring systems, resistance combinations

## Abstract

A joint comparative regional analysis of different resistance combinations across human and veterinary medicine has not been previously conducted in Germany. This study analyses 16 resistance combinations from four antibiotics in *E. coli* from different human and food-producing animal populations in three German regions: East, North West and South West. The *E. coli* data were collected from the three national surveillance and monitoring systems for antimicrobial resistance (AMR) bacteria in humans (ARS), food-safety (Zoonosis Monitoring) and animal pathogens (GE*RM*-Vet) from January 2014 to December 2017. Analyses were performed using cluster analysis (hierarchical clustering, average linkage) in R. We included data from 537,215 *E. coli* isolates from human clinical isolates, from clinical as well as non-clinical isolates from food-producing animals and from food. The majority of the data originated from the North West region. There were two main clusters built on 54 different human and animal populations. We observed close similarities of resistance combinations in human isolates from the different regions within the same human populations from outpatient cares, general wards and ICUs. These resistance combinations clustered separately from non-clinical isolates from broilers, turkeys, cattle and pigs; except for some of clinical isolates from these populations which clustered closely to isolates from human populations. Frequently, the resistance combinations in *E. coli* isolates from farms clustered closely to the resistance combinations in isolates from slaughterhouses from broilers and turkeys over all regions. However, the resistance combinations in *E. coli* isolates from retail meat populations tended to cluster separately within their respective populations in between all regions.

## Introduction

In Germany, regional differences in the prevalence of antimicrobial resistant (AMR) bacteria have previously been observed. In humans, the occurrences of carbapenem resistant *Klebsiella pneumoniae* and *Acinetobacter baumanii* (1, 2), vancomycin resistant *Enterococcus faecium* (3), methicillin resistant *Staphylococcus aureus* (MRSA) (4), and uropathogenic *Escherichia coli* (5) varied between German regions. Such regional differences have also been observed in e.g., occurrence of MRSA in dairy cows in Germany (6). Although regional differences in Germany were previously studied for the resistance patterns in different human and food-producing animal pathogens, a comparative regional analysis of resistance combinations between human and different food-producing animal populations has not previously been conducted. This interregional comparison analysis is important, since regional differences in resistance of bacteria from humans and different animal populations might be associated with exchange of bacteria between humans and animal populations within region (7). Therefore, the goal of this study is to compare the resistance patterns in *E. coli* isolates from humans and different food-producing animal populations considering four antibiotics—ampicillin (AMP), cefotaxime (CTX), ciprofloxacin (CIP), and gentamicin (GEN)—between three different regions of Germany: East, North West and South West. It will challenge the hypothesis that similar patterns of resistance are observed in different populations of the same region along with differences in patterns between regions in Germany.

## Methods

### Data Selection

We divided Germany into three regions based on the population structure of different animal populations as previously described by Tenhagen et al. (6). The “East” region is characterized by a low number of herds with a large herd size and an overall low regional animal density. It included Berlin, Brandenburg, Mecklenburg Western Pomerania, Saxony-Anhalt, Saxony, and Thuringia. The “North West” region is characterized by a high number of animal populations with a high regional animal density and a smaller, but still rather large, herd size compared to region East. It includes Schleswig Holstein, Lower Saxony, North Rhine Westphalia, Bremen, and Hamburg. The “South West” region, which is characterized by a high number of animal populations with a high regional animal density and a comparatively small herd size, represents Bavaria, Baden-Wuerttemberg, Hesse, Rhineland Palatinate, and Saarland.

The data for this study were collected between January 2014 and December 2017. For the same study period, we previously studied similarities in resistance patterns of *E. coli* isolates from different human and animal populations for the whole of Germany (8). Data on human isolates originated from the *Antibiotika Resistenz Surveillance* (ARS) system (9). All data on non-clinical *E. coli* isolates from food-producing animals and food came from the Zoonosis Monitoring that were collected in Germany (10). There were no non-clinical isolates collected from cattle from farms during this study period. Data on clinical isolates from animals were taken from GE*RM*-Vet (11), the system for the monitoring of resistance in animal pathogens in Germany. Detailed information on these systems was summarized in a previous study (8).

Four frequently tested antibiotics in ARS, Zoonosis Monitoring and GE*RM*-Vet - ampicillin, cefotaxime, ciprofloxacin, and gentamicin—were included. The sixteen resistance combinations to these four antibiotics—were calculated using the permutation function. Detailed information on the inclusion criteria has previously been described (8). This study included *E. coli* isolates from humans, broilers, turkeys, pigs, and cattle populations stratified by their origins: for human populations outpatient care (A), intensive care unit (ICU), general ward (GW); for non-clinical animal populations, farm (F), slaughterhouse (S) and retail (R) and for clinical animal populations (C) (8) (Supplementary Table 1).

For the purpose of cluster analysis, each population was split into three different regional sub-populations: East, North West and South West. In total, there were 54 regionally stratified populations derived from the in total 18 populations for human and different animal populations in each region. All 54 regional populations were included in one model. For the analysis several pig populations (growers, sows, fattening pigs, piglets and weaners) and cattle populations (bovines <1 year and dairy cows) had to be grouped into “pigs” and “cattle” respectively to account for small sample sizes in the sub-populations. Eleven non-clinical animal populations and two clinical animal populations from the national model (9) were excluded from this study on account of too few isolates in the regions (Supplementary Table 2).

### Statistical Analysis

This study used cluster analysis to analyze similarities of sixteen resistance combinations between different human and animal populations in three German regions. Cluster analysis on resistance combinations was performed with the hierarchical clustering using Euclidian distance and the average linkage. This method was adapted from the previous study on statistical methodology for analysis of multi-drug resistant bacteria by Jasper et al. (12). For the purpose of our study, the step “multiple correspondence analysis” to reduce number of resistance combinations was excluded. This was not necessary for our datasets since there were only 16 resistance combinations built from four antibiotics. Similar to the previous study, average linkage was chosen because of inclusion of all study populations. The number of clusters was determined visually by the silhouette plot (8) and elbow method. In this study, we defined main clusters (Cluster) and sub-clusters (SC) to support the readers for differentiating the populations in the cluster visualization. All analyses were conducted with R 3.5.1 (Rstudio 1.1.442). The same R-packages as previously described were used (8).

## Results

### Descriptive Analysis

Data were collected from 537,215 *E. coli* isolates from ARS, Zoonosis Monitoring, and GE*RM*-Vet. The data extraction from each system has been previously described (8). The number of farms, animals, animals per farms, human populations, participating hospitals, and general practices in ARS systems, and numbers of *E. coli* isolates from different systems for different populations in each study region are summarized in Supplementary Table 3. After the exclusion of non-target populations for this study (Supplementary Table 1), 327,416 isolates were included in this study. Out of these isolates, 320,555 isolates (98%) originated from human populations: 30,328 isolates from ICU (9.3%), 197,521 isolates from general ward (60.3%) and 92,706 isolates (28.3%) from outpatient care; 4,298 isolates were non-clinical isolates (1%) from food-producing animals and food, and 2,563 isolates (1%) were clinical isolates from food-producing animals (Supplementary Table 3).

### Cluster Analysis

**Figure 1:**
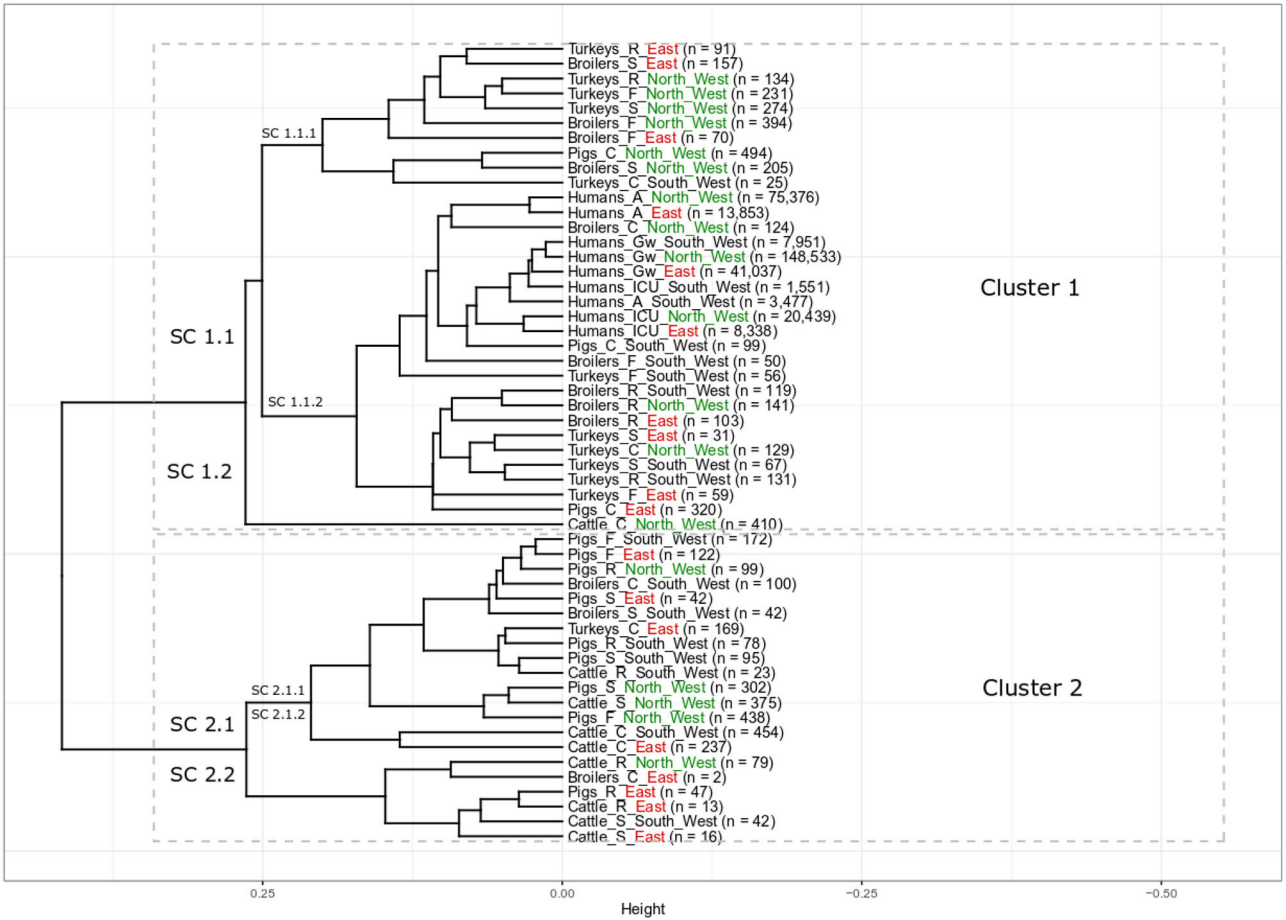
Results of cluster analysis of the resistance combinations in *E. coli* isolates from 18 grouped populations from different human and food-producing animal populations between 2014 and 2017. There are two main clusters with eight sub-clusters (SC). Human population: A: isolates from outpatient care, Gw: isolates from general ward and ICU: isolates from intensive care unit. Animal population: F: isolates from farms, S: isolates from slaughterhouse and R: isolates from retail, C: clinical isolates from animals. n is the total number of included isolates. For better regional separation, the regions are additionally color coded with North West in green, East in red and South West in black.

The elbow and silhouette methods suggested two main clusters (Supplementary Figure 1). Cluster 1 includes the majority of all populations (33 populations, 61%) including all nine populations of clinical isolates from humans, 17 populations of non-clinical animal isolates from food-producing animals and foods, and seven populations of clinical isolates from food-producing animals. The second cluster contains 21 different food-producing animal populations (39%) with 16 populations of non-clinical animal isolates from food producing animals and foods and five populations of clinical isolates from food-producing animals (Figure 1).

All isolates from the different **human populations** from all three different regions clustered next to each other in one sub-cluster (Figure 1, Cluster 1, SC 1.1.2). Within SC 1.1.2 there were two slightly separated groups. One of those contained all human isolates, while the other contained mainly poultry isolates. Isolates from humans in general wards from the three regions clustered closely together (Humans_Gw_South_West, Humans_Gw_North_West, and Humans_Gw_East). Isolates from ICUs were their closest neighbor. The isolates from humans in outpatient care facilities from the North West (Humans_A_North_West) and the East (Humans_A_East) clustered closely together separated only slightly from the other human isolates. Clinical isolates from two food-producing animal populations clustered in the same part of the sub-cluster with the isolates from humans: clinical isolates from broilers in the North West (Broilers_C_North_West) and clinical isolates from pigs from the South West (Pigs_C_South_West). At a slightly larger distance, this part of the sub-cluster contains also isolates from broilers and turkeys on farms in the South West (Broilers_F_South_West and Turkeys_F_South_West).

**The isolates from broilers** on farms in the North West (Broilers_F_North_West) and the East (Broilers_F_East) clustered together in one sub-cluster that predominantly contained poultry isolates (Cluster 1, SC 1.1.1). It also included the respective regional isolates collected at slaughter. The isolates from broilers on farms in the South West (Broilers_F_South_West) clustered (Cluster 1, SC 1.1.2) separately from broilers from the same region at the slaughterhouse (Cluster 2, SC 2.1.1). Isolates from broiler meat at retail from the three different regions all clustered together (Cluster 1, SC 1.1.2). In contrast, clinical isolates from broilers from the three regions clustered separately from each other in different main clusters (SC 1.1.2, 2.1.1 and 2.2 respectively).

Most isolates from **turkeys** clustered in two different sub-clusters in cluster 1. All non-clinical isolates from turkeys in the North West on farms, in slaughterhouses and at retail clustered together in SC 1.1.1 (Turkeys_F_North_West, Turkeys_S_North_West, and Turkeys_R_North_West). The SC 1.1.2 contains all non-clinical isolates from turkeys in the South West (i.e., farm, slaughterhouse, retail). It also contained isolates from farms and from the slaughterhouses from the East (Turkeys_F_East and Turkeys_S_East). Clinical isolates from turkeys from the three regions all appeared in different subclusters (Turkeys_C_North_West in SC 1.1.2, Turkeys_C_South_West in SC 1.1.1 Turkeys_C_East in SC 2.1.1).

Nearly **all non-clinical isolates from pigs** clustered together in Cluster 2, SC 2.1.1. Only isolates from pork at retail in East (Pigs_R_East) clustered separately (SC 2.2). They all clustered separately from the isolates from the “human cluster” (SC 1.1.2). In SC 2.1.1, the non-clinical isolates from pigs clustered together with clinical isolates from broilers in the South West (Broilers_C_South_West) and turkeys in the East (Turkeys_C_East). It clustered also together with isolates from two cattle populations (Cattle_R_South_West and Cattle_S_North_West). The clinical isolates from pigs clustered separately from the non-clinical isolates from pigs. Clinical isolates from pigs from two different regions (Pigs_C_South_West and Pigs_C_East) clustered in the same sub-cluster with the isolates from humans (SC 1.1.2). The clinical isolates from pigs from the North West (Pigs_C_North_West) clustered in SC 1.1.1 and were the only non-poultry isolates in that cluster.

**The isolates from cattle** clustered in both clusters, one population in Cluster 1 and eight populations in Cluster 2. Interestingly, all clinical isolates from cattle clustered separately from the isolates from other food-producing animal populations. The clinical isolates from cattle from the North West (Cattle_C_North_West) clustered alone in one sub cluster (SC 1.2), those of the other regions were alone in SC 2.1.2. The isolates from cattle in slaughterhouses in the South West and the East, (Cattle_S_South_West and Cattle_S_East) clustered together in the same sub-cluster (Cluster 2.2) that also included the isolates of bovine meat from the East. The isolates from these two regions clustered separately from the isolates from the slaughterhouse and from meat at retail in the North West (Cluster 2, SC 2.1.1). The isolates from bovine meat at retail were separated according to region: Cattle_R_South_West in SC 2.1.1, Cattle_R_North_West and Cattle_R_East in SC 2.2.

## Discussion

This study compared resistance combinations in *E. coli* from different populations in three German regions. It built upon a previous study (8) to investigate potential regional associations of AMR bacteria between isolates from different human and food-producing animal populations. As observed in the earlier study, all human isolates from different health care facilities clustered together. This was confirmed regardless of the different regions of origin. However, the different levels of the health care facilities (outpatient, general ward and intensive care) tended to cluster together across regions indicating a stronger association of the level of health care as compared to the regional stratification. This effect was less pronounced with the isolates from outpatient care where isolates from the North West and the East were slightly separated from those from the South West. This separation remains however unclear and should be considered in the further comparative analyses between German regions. As the level of antimicrobial use tends to differ between the different levels of health care facilities, more differences between samples from these subpopulations might have been expected. However, detailed data on antimicrobial use in these populations in Germany are not available and therefore cannot be used in the analysis. This should be addressed in future studies. The close similarities of human isolates reported from this study confirmed previous study that was carried out addressing ESBL/AmpC genes specifically (13). In addition to that, a population-based study in Netherland reported that most of ESBL producing bacteria in the general population of the Netherlands was probably originated from other human populations (7).

Closer regional associations were seen for some of the food-producing animal populations. Most poultry populations were in cluster 1 and often isolates from farms clustered in the same sub-cluster with isolates from the slaughterhouses from the same region. These animals will frequently be slaughtered in the same region that they are raised in to avoid long transport. An exception was observed for the South West, where broilers at farm and at slaughter were in two different main clusters (1 and 2, respectively). All isolates from pigs at farm and pigs at slaughter from the same region were observed in the same sub-cluster (2.1). For cattle, this association could not be studied as no non-clinical isolates had been collected at the farm level in the period. The non-clinical food-producing animal isolates coming from farms and slaughterhouses were collected in the framework of food-safety monitoring in Germany. These isolates are mandatorily collected from the German domestic primary productions, i.e., excluding slaughter batches from neighboring countries that may have different levels of antimicrobial resistance (14, 15).

In contrast, all samples from broiler meat at retail from all regions were in the same cluster as closest neighbors indicating that isolates from broiler meat sold in different parts of the country share similar AMR patterns. This was not observed for turkey meat (two populations in SC 1.1.1, one in 1.1.2), pigs (two in SC 2.1.1, one in 2.2) or cattle (one in SC 2.1.1 and two in 2.2). Retail samples were not restricted to domestic production and therefore may include products originating for other EU-Member states or even third countries. Moreover, some isolates on meat may originate from contamination at slaughter or during further processing. This might explain some differences between the slaughterhouse and the retail level. Proximity of broiler meat isolates from different regions might indicate trade of broiler meat across the country, irrespective of region. This indicated a more regional trade of turkey, pig and bovine meat (15). In line with that, in two regions turkey meat clustered closely with turkeys at slaughter. However, trade data to confirm this are not available.

For the regional model, we gathered the isolates from different cattle and pig populations to the species level, to account for the small sample sizes in the three regions (Supplementary Table 2). The monitoring programs in the food chain are not designed for regional stratification but for national estimates. Therefore, samples are assigned to regions proportionate to the size of the respective population in the region to better reflect the national population. In consequence, sample sizes may be small in some regions, if most of the food-producing animals are housed in other regions.

In this study, the clinical isolates from cattle (Cattle_C) predominantly contain the clinical isolates from bovines <1 year. In the previous national model, the clinical isolates from bovine <1 year clustered separately in one main cluster due to their higher relative frequencies of all resistance combinations than other isolates from cattle populations (8). In this study clinical isolates from cattle in the North West, also formed a cluster of their own and those from the East and the South West formed a separate sub-cluster, indicating that resistance patterns in clinical isolates from cattle differ substantially from the other bacterial populations and between the North West where most of the veal calves are raised and the South West and East.

This study addresses the similarity of resistance combinations of AMR bacteria between human and different animal populations. These data were obtained in three different systems and it could be speculated that differences in the resistance combinations reflect differences in the systems. However, we recently retested isolates from medical laboratories using broth microdilution as used in the animal and food isolates. We found a good agreement of the results (16). As previously described in the national model (8), the close similarities of resistance combinations between clinical isolates from different animal populations and clinical isolates from human in different levels of health care facilities were also observed in this study. Additionally, we observed close similarities of resistance combinations between pigs- and poultry populations in different German regions. These similarities were also reported in the national model and earlier study that reported high ampicillin resistance in pigs and poultry populations (8, 17). However, the transmission of AMR bacteria between humans and different animal populations in different German regions remains complex and cannot be unraveled with our datasets. The data was mostly collected in the North West, both for the different human populations and different food-producing animal populations. This is in line with the high density of livestock production in the North West (18) and high number of participating health care facilities in the human surveillance system (1–3) (Supplementary Table 3). We studied only resistance to four antibiotics that were routinely included in all three monitoring and surveillance systems in one country. The situation and the clustering of isolates from the different populations may differ substantially in other countries with different treatment patterns and level of antimicrobial use as indicated by data on antimicrobial consumption in food-producing animals and humans provided by the European institutions (19, 20). A number of antimicrobials had to be excluded as they were only tested in one or two of the studied systems. This calls for a better harmonization of resistance testing in the one health context. Due to the structure of surveillance and monitoring datasets the regional analyses are limited. Additional indicators such as trade (21), and animal movement (22) should be considered in further studies. Further investigations on the food-chain network between the European countries will support as well further explanations on the variation of resistance combinations between the countries, as these countries have different regulations on monitoring systems (23).

To the best of our knowledge, this study is the first study on regional analyses of resistance combinations in different human and food-producing animal populations in Germany. Regional cluster analysis with the routine phenotypical AMR data underlines the complexity of the relationship between AMR in human and different animal populations. It also underlines that the human isolates tend to cluster together and separate from most of the healthy food-producing animal isolates. Further regional analyses should consider additional information such as structures of counties, e.g. rural and urban, other relevant antibiotics, and information on trade and animal movement in the country.

## Data Availability Statement

The aggregated data supporting the conclusions of this article will be made available by the authors, without undue reservation.

## Author Contributions

BS and B-AT conceptualized the study. B-AT, TE, and HK provided the datasets. BS analyzed the datasets and wrote the manuscript. B-AT, TE, and HK read and reviewed the manuscript. All authors have read and agreed on submission of the manuscript.

## Funding

This study was funded by the German Federal Institute for Risk Assessment within German One Health Initiative (GOHI) (Grant No. 1322-687).

## Conflict of Interest

The authors declare that the research was conducted in the absence of any commercial or financial relationships that could be construed as a potential conflict of interest.

## Publisher's Note

All claims expressed in this article are solely those of the authors and do not necessarily represent those of their affiliated organizations, or those of the publisher, the editors and the reviewers. Any product that may be evaluated in this article, or claim that may be made by its manufacturer, is not guaranteed or endorsed by the publisher.
