# Supplementary material for: A Joint Regional Analysis of Resistance Combinations in Escherichia coli in Humans and Different Food-Producing Animal Populations in Germany Between 2014 and 2017
Source: Front Public Health. 2022 Jun 9;10:823613. doi: 10.3389/fpubh.2022.823613 (PMC9218088; doi:10.3389/fpubh.2022.823613)
Supplement: Supplementary file 1 [file Table_1.DOCX]

Supplementary Material

# Supplementary Tables

Supplementary Table 1. Grouped Populations from different origins.

| **Isolate type** | **Origin** | **Populations** | **Grouped** |
| --- | --- | --- | --- |
| **Non-clinical food-producing animal isolates** (11 grouped populations, incl. 4 grouped food populations) | Farm (F) | Broilers, F | Broilers_ F |
|  |  | Broilers Conv, F | Broilers_F |
|  |  | Broilers Org, F | Broilers_F |
|  |  | Turkeys, F | Turkeys_F |
|  |  | Growers <50 kg, F | Pigs_F |
|  |  | Weaners, F | Pigs_F |
|  |  | Sows, F | Pigs_F |
|  | Slaughter (S) | Broilers, S | Broilers_S |
|  |  | Turkeys, S | Turkeys_S |
|  |  | Bovines <1year, S | Cattle_S |
|  |  | Fattening pigs, S | Pigs_S |
|  | Retail (R) | Broiler meat, R | Broilers_R |
|  |  | Turkey meat, R | Turkeys_R |
|  |  | Bovine meat, R | Cattle_R |
|  |  | Pork, R | Pigs_R |
|  |  | Raw sausages, R | Pigs_R |
| **Clinical food-producing animal isolates**  (4 grouped populations) | Farm/Veterinary practice | Piglets, C | Pigs_C |
|  |  | Growers, C | Pigs_C |
|  |  | Pigs, C | Pigs_C |
|  |  | Sows, C | Pigs_C |
|  |  | Broilers, C | Broilers_C |
|  |  | Turkeys, C | Turkeys_C |
|  |  | Bovines <1year, C | Cattle_C |
|  |  | Cattle, C | Cattle_C |
|  |  | Dairy cows, C | Cattle_C |
| **Clinical human isolates** (3 grouped populations) | Outpatient (A) | Humans, A | Humans_A |
|  | General Ward (GW) | Humans, Gw | Humans_Gw |
|  | Intensive Care Unit (ICU) | Humans, ICU | Humans_ICU |

Supplementary Table 2. List of excluded datasets

| **Isolate type** | **Origin** | **Populations** |
| --- | --- | --- |
| **Non-clinical animal isolates** (11 populations, incl food and wild) | Farm (F) | Laying hens, F |
|  |  | Breeder chickens, F |
|  |  | Bovine milk, Conv, F |
|  |  | Bovine milk, Org, F |
|  |  | Bivalves, F |
|  | Retail (R) | Venisons, R |
|  |  | Shrimps, R |
|  |  | Table eggs, R |
|  |  | Bivalves, R |
|  | Wild/Game (W) | Roe deer hunted, W |
|  |  | Wild boar hunted, W |
| **Clinical animal isolates** (2 populations) | Farm/veterinary practices | Laying hens, C |
|  |  | Small animals, C |

Supplementary Table 3. The origins with their respective population data in the regions in Germany and the distribution of the included *E. coli* isolates among the three study regions. NA: *E. coli* isolates were not collected for cattle on farms in Zoonosis Monitoring from 2014 and 2017

|  | | | | | | | |
| --- | --- | --- | --- | --- | --- | --- | --- |
| **Study Region** | **Destatis (2016/2017)** | | | **Zoonosis monitoring  (2014-2017)** | | | **GERM-Vet  2014-2017** |
|  | **No. of Farms** | **No. of food-producing animals** | **Animals/ farm** | **No. of *E. coli* isolates from Farm** | **No. of *E. coli* isolates from Slaughter-house** | **No. of *E. coli* isolates from Retail** | **No. of clinical *E. coli* isolates** |
| **Broilers** | | | | | | | |
| East | 318 | 17,138,591 | 53,894.9 | 70 | 157 | 103 | 2 |
| North-West | 1556 | 68,920,342 | 44,293.3 | 394 | 205 | 141 | 124 |
| South West | 1456 | 7,726,826 | 5,306.9 | 50 | 42 | 119 | 100 |
| **Turkeys** | | | | | | | |
|  |  |  |  |  |  |  |  |
| East | 261 | 3,491,637 | 13,377.9 | 59 | 31 | 91 | 169 |
| North-West | 674 | 6,863,119 | 10,182.7 | 231 | 274 | 134 | 129 |
| South West | 867 | 1,981,061 | 2,285 | 56 | 67 | 131 | 25 |
|  |  |  |  |  |  |  |  |
| **Pigs** | | | | | | | |
| East | 1,000 | 4,263,300 | 4,263.3 | 122 | 42 | 47 | 320 |
| North-West | 14,100 | 1,7501,400 | 1,241.2 | 438 | 302 | 99 | 494 |
| South West | 8,500 | 5,814,800 | 684.1 | 172 | 95 | 78 | 99 |
|  |  |  |  |  |  |  |  |
| **Cattle** | | | | | | | |
| East | 21,643 | 2,210,477 | 102.1 | NA | 16 | 13 | 237 |
| North-West | 45,578 | 5,125,507 | 112.5 | NA | 375 | 79 | 410 |
| South West | 76,380 | 4,945,211 | 64.7 | NA | 42 | 23 | 454 |
|  |  |  |  |  |  |  |  |

| **Humans** | | | | | | | |
| --- | --- | --- | --- | --- | --- | --- | --- |
| **Study Region** | **Destatis 2020*^(11)^** | **ARS (2014-2017)** | | | | |  |
|  |  | **No. of participating Hospitals** | **No. of participating General Practices** | **No. of *E. coli* isolates from Intensive Care Unit (ICU)** | **No. of *E. coli* isolates from General Ward** | **No. of *E. coli* isolates from Outpatient care** |  |
| East | 16,199,653 | 11 | 75 | 8,338 | 41,037 | 13,853 |  |
| North-West | 31,373,057 | 2,236 | 109 | 20,439 | 148,533 | 75,376 |  |
| South West | 35,594,001 | 1,045 | 71 | 1,551 | 7,951 | 3,477 |  |

*2011 Census

# Supplementary Figures


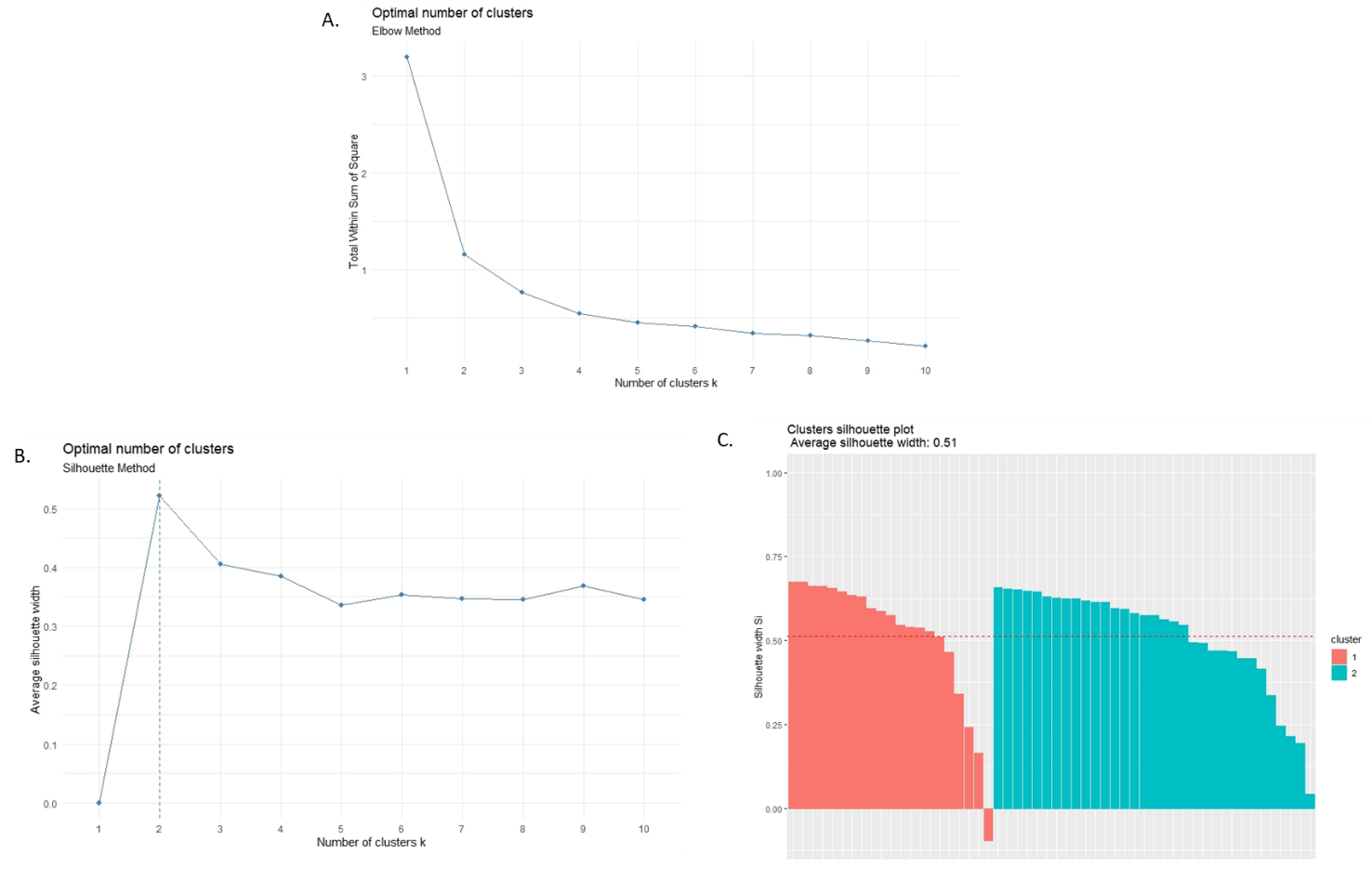


Supplementary Figure 1. Determination of number of clusters with elbow (A) and silhouette methods (B and C).
